# Bilayer Hydrogel Composed of Elastin-Mimetic Polypeptides as a Bio-Actuator with Bidirectional and Reversible Bending Behaviors

**DOI:** 10.3390/molecules28135274

**Published:** 2023-07-07

**Authors:** Rui Kamada, Hiromitsu Miyazaki, Jose Isagani B. Janairo, Yoshiro Chuman, Kazuyasu Sakaguchi

**Affiliations:** 1 Laboratory of Biological Chemistry, Department of Chemistry, Faculty of Science, Hokkaido University, Sapporo 060-0810, Japan; kamadar@sci.hokudai.ac.jp (R.K.);; 2 Department of Biology, College of Science, De La Salle University, Manila 0922, Philippines; jose.isagani.janairo@dlsu.edu.ph; 3 Laboratory of Biological Chemistry, Department of Chemistry, Faculty of Science, Niigata University, Niigata 950-2181, Japan; chuman@chem.sc.niigata-u.ac.jp

**Keywords:** bilayer hydrogel, intelligent gel, peptides, protein engineering, soft actuator, salt-responsive, thermos-responsive

## Abstract

Biologically derived hydrogels have attracted attention as promising polymers for use in biomedical applications because of their high biocompatibility, biodegradability, and low toxicity. Elastin-mimetic polypeptides (EMPs), which contain a repeated amino acid sequence derived from the hydrophobic domain of tropoelastin, exhibit reversible phase transition behavior, and thus, represent an interesting starting point for the development of biologically derived hydrogels. In this study, we succeeded in developing functional EMP-conjugated hydrogels that displayed temperature-responsive swelling/shrinking properties. The EMP-conjugated hydrogels were prepared through the polymerization of acrylated EMP with acrylamide. The EMP hydrogel swelled and shrank in response to temperature changes, and the swelling/shrinking capacity of the EMP hydrogels could be controlled by altering either the amount of EMP or the salt concentration in the buffer. The EMP hydrogels were able to select a uniform component of EMPs with a desired and specific repeat number of the EMP sequence, which could control the swelling/shrinking property of the EMP hydrogel. Moreover, we developed a smart hydrogel actuator based on EMP crosslinked hydrogels and non-crosslinked hydrogels that exhibited bidirectional curvature behavior in response to changes in temperature. These thermally responsive EMP hydrogels have potential use as bio-actuators for a number of biomedical applications.

## 1. Introduction

Stimulus-responsive hydrogels, which induce volume phase transitions in response to small changes in the external environment, such as temperature, pH, solvent composition, electric field, light, and the introduction of specific molecules, have attracted great attention from the perspective of potential biomedical applications [1,2,3]. Owing to their flexible behavior and low-friction properties, this class of material is highly relevant to various fields, such as drug delivery systems, separation materials, sensors, actuators, and biomimetic materials. Poly(N-isopropylacrylamide) gel (PNIPAM gel) is the best known example of a temperature- and stimulus-responsive hydrogel and exhibits volume phase transition behavior at approximately 34 °C [4,5]. PNIPAM gel has been investigated in a wide range of practical applications, including drug delivery systems [6,7] and as an actuator. In the case of its use as an actuator, a microfluidic device, which can adsorb and release proteins using PNIPAM film [8] and through the temperature-dependent control of cultured cell adhesion and desorption [9,10], has been reported. Additionally, their application to bio-gel actuators and artificial muscles has been attempted using polyvinyl methyl ether gels (PVME gels), which exhibit temperature phase transition behavior similar to that of PNIPAM gels [11].

Since stimulus-responsive gels are envisioned to be used mostly in biomedical applications, using natural polymers or biologically derived building blocks possess intrinsic advantages. The advantage of using natural or biologically derived polymers include high biocompatibility, easy biodegradability, and low toxicity [12]. However, most of the stimulus-responsive gels reported so far, including PNIPAM gels, are composed of chemically synthesized polymers [2,13]. Thus, developing materials composed of biologically derived building blocks is important because they have the potential to have a very important impact on the advancement of material sciences for treating human diseases. Among the different biomolecules available to be used as building blocks for the creation of polymers, proteins are attractive due to their versatility and functionality.

Not surprisingly, a number of different proteins have already been used for the fabrication of medically relevant materials, and these include collagen, gelatin, keratin, and zein [14]. Another protein that is very attractive for use in polymer synthesis is elastin, a key protein present in flexible tissues in higher vertebrates that is responsible for providing them with their elasticity and recoil. One of the major repeating sequences in elastin is the pentapeptide repeat sequence (Val-Pro-Gly-Xaa-Gly)n (Xaa excludes Pro), which exhibits reversible phase transition behavior [15]. That is, below the phase transition temperature, they are highly soluble in water, but above this temperature, they undergo reversible phase separation to form insoluble coacervates.

This coacervation behavior of EMPs is thought to be induced by the loss of hydrogen bonds in the reaction and the increase in hydrophobic interactions between EMPs with increasing temperature [16,17]. Furthermore, EMPs are known to have extremely low antigenicity and toxicity to living organisms [18]. EMPs are peptides, which means that not only can their functional groups be easily modified, but they are also generally compatible with the environment due to the fact that they are generally biodegraded with little or no difficulty. Therefore, EMPs have the potential to be extremely useful as functional materials whose structure and function can be reversibly controlled by controlling external stimuli such as temperature and salt concentration. Although elastin has not been used much in hydrogel production, it has been reported that EMPs can be used for thermally targeted drug delivery and as skin wound healing scaffolds [19,20]. Thus, the cross-linking of EMPs with such properties into hydrogels is expected to lead to the development of stimulus-responsive hydrogels that are more compatible with living organisms and the environment.

In this study, EMPs were cross-linked to polymer chains to develop thermo-responsive hydrogels that exhibit reversible swelling/shrinking behavior in response to temperature change, and to develop thermo-responsive functional hydrogels as their application.

## 2. Results and Discussion

### 2.1. Coacervation Activity of EMP Monomer and Acryl-EMP Monomer

**Figure 1:**
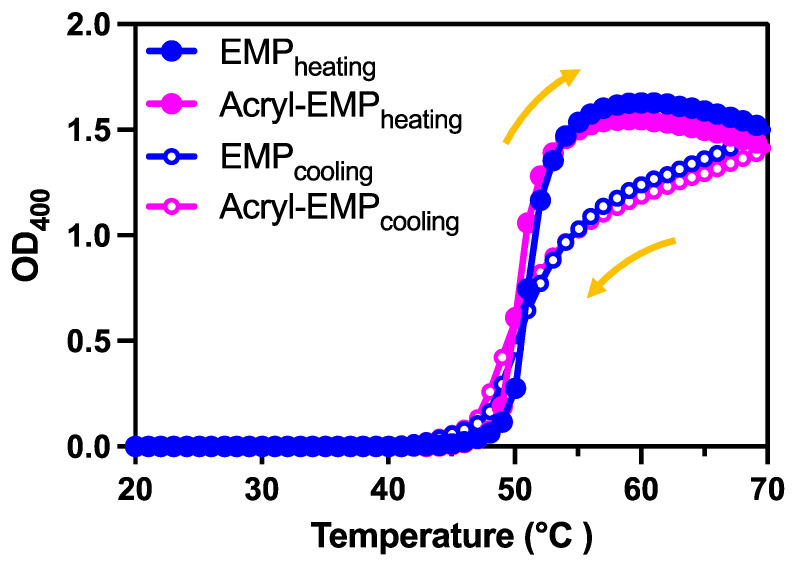
Optical density of EMP (blue) and acryl-EMP (magenta) as a function of temperature. Optical density of EMP (20 µM) and acryl-EMP (20 µM) in response to temperature during a heating phase (20 °C to 70 °C, closed circle), and then, a cooling phase back to 20 °C (open circle). The left arrow indicates heating, and the right arrow indicates cooling.

The coacervation properties of Acryl-EMP and EMP were determined by monitoring their temperature-dependent absorbance at 400 nm (OD_400_) (Figure 1). EMP at a concentration of 20 µM exhibited a sharp increase in turbidity (OD_400_) as the temperature was increased from 48 °C to 54 °C, and the turbidity returned to baseline when the temperature was returned to below roughly 40 °C. This result indicates that EMP has reversible coacervation property. Acryl-EMP displayed a coacervate property similar to EMP, wherein the transition temperature was around 45 °C, suggesting that the conjugation of EMP with acrylic acid had virtually no effect on its coacervation property.

### 2.2. Preparation of Hydrogels with Acryl-EMP

**Figure 2:**
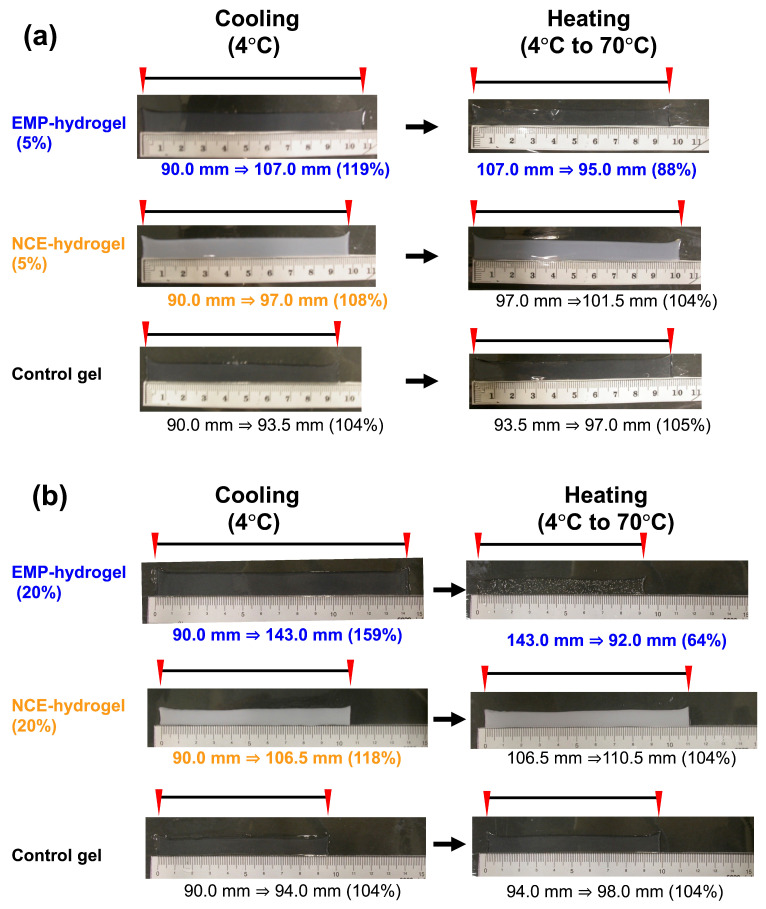
Photographs of the EMP hydrogel treated at different temperatures: (**a**) 5% EMP hydrogel, (**b**) 20% EMP hydrogel.

We prepared EMP-conjugated polyacrylamide gels by polymerizing acryl-EMP with acrylamide (5 and 20% acryl-EMP). In order to analyze the effect of temperature change on the crosslinked EMP hydrogels (referred to as EMP hydrogels), several types of hydrogel were prepared. The first type was a 5%-non-crosslinked EMP (NCE) hydrogel (5%-NCE hydrogels), in which EMP was not crosslinked with acrylamide and the control hydrogel was also prepared without the presence of the EMP. The change in the lengths of the three different hydrogels with respect to changes in temperature was determined (Figure 2). Incubation at 4 °C induced a 19% increase in length (90 to107 mm) in the 5%-EMP hydrogel. In contrast, only an 8% change was observed for the 5%-NCE hydrogel (90 to 97 mm) and only a 4% change was observed for the control hydrogel (90 to 93.5 mm) at 4 °C. In addition, when the gels were incubated at 70 °C, the control and 5%-NCE hydrogels showed roughly similar increases in gel length (97 to 101.5 nm and 93.5 to 97 nm), whereas the 5%-EMP hydrogels showed a 13% decrease in gel length (107 to 95 nm) (Figure 2a). These results highlight that the crosslinked EMP hydrogels are more sensitive to the temperature of its environment compared to the non-crosslinked and control gels. Moreover, the results indicate that the EMP hydrogels crosslinked with the acryl-EMP modification of the N-terminal amino group exhibited swelling behavior at low temperatures and shrinking behavior at high temperatures. These results demonstrate that crosslinking acryl-EMP with polymer chains represents a promising approach to developing thermo-responsive hydrogels that can reversibly swell and shrink depending on the nature of the thermal stimulus.

Next, the temperature-dependent swelling behaviors of a 20% EMP hydrogel and a 20% NCE hydrogel were compared (Figure 2b). As shown in Figure 2b, the 20% EMP hydrogel showed a 59% increase in length (90 to 143 mm) after incubation at 4 °C. In contrast, the control hydrogel displayed roughly a 4% increase in length (90 versus 94 nm) and the 20% NCE hydrogel showed an 18% increase in length (90 to 106.5) at 4 °C. With a similar, but more dramatic, result to what was observed with the 5% EMP hydrogel, incubation at 70 °C decreased the length of the 20%-EMP hydrogel by 64% (143 to 92 mm), whereas a slight increase in length was observed with the NCE hydrogel (106.5 to 110.5 mm) and the control gel (94 to 98 mm) at 70 °C. From these results, we determine that the swelling ratio of the 5% EMP hydrogel was lower than that of the 20% EMP hydrogel, which indicates that the swelling property of the EMP hydrogels can be controlled by the amount of acryl-EMP added in the hydrogel. Thus, the temperature-dependent swelling and shrinkage of the hydrogels can be attributed to the EMP crosslinked with acrylamide. Interestingly, the NCE hydrogel was strongly opaque both below and above its lower critical solution temperature (LCST), suggesting that EMP in the NCE hydrogel formed a coacervate. In contrast, the EMP hydrogels were transparent at all temperatures, which we hypothesize is due to the acrylamide gel networks preventing EMP aggregation in the gels.

### 2.3. Property of Temperature-Responsive EMP Hydrogel

**Figure 3:**
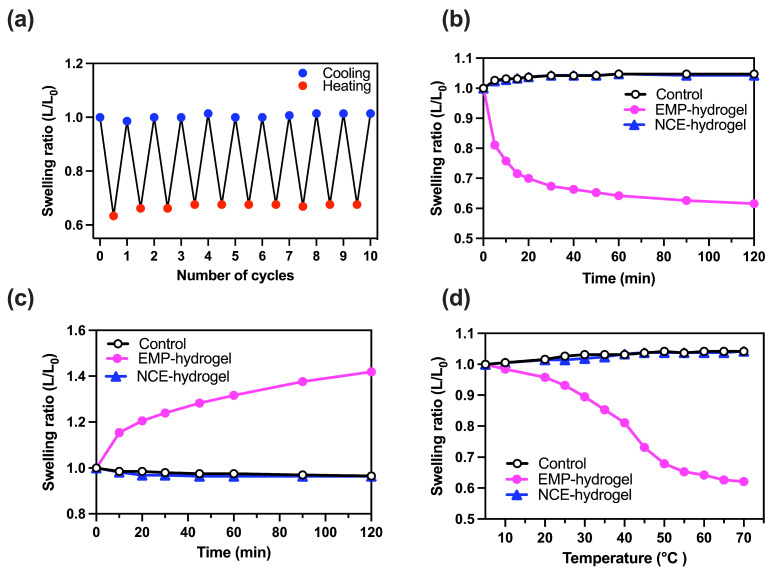
Swelling/shrinking response of 20% EMP hydrogel to temperature. (**a**) Repeatability on the swelling ratio (L/L_0_) of EMP hydrogel upon either heating (blue dots) or cooling (red dots) of the buffer between 70 °C and 5 °C. (**b**,**c**) Swelling ratio of the 20% EMP hydrogel (magenta closed circles), 20% NCE hydrogel (blue triangles), and control hydrogel (black open circles) measured at a particular time following either heating ((**b**), 70 °C) or cooling ((**c**), 5 °C). (**d**) Temperature dependence of the swelling ratio of hydrogels after 2 h incubation at temperatures between 5 °C and 70 °C.

To further analyze the temperature-dependent swelling/shrinking properties of the EMP hydrogel, we determined the reversibility of the thermal response of the 20% EMP hydrogel. This was performed by maintaining the temperature first at 70 °C for 30 min, and then, at 5 °C for 10 h. This temperature variation was repeated for 10 cycles (Figure 3a). The results show that the EMP hydrogel swelled and shrank reversibly in response to these cyclical high–low temperature changes, and there was no notable change in the swelling/shrinking properties of the gel even after the 10 cycles.

The temporal swelling-and-shrinking ratio of the 20% EMP hydrogel and the 20% NCE hydrogel was measured in Tris-HCl pH 8.8 buffer (Figure 3b,c). The swelling/shrinking properties were first monitored after placing the 20% EMP hydrogel into the buffer at 70 °C (Figure 3b), where it was found to dramatically start decreasing in length within the first 30 min, and the decrease in the swelling ratio was more or less finished within 2 h. Next, the 20% EMP hydrogel was placed into the buffer at 5 °C (Figure 3c), where it was found to increase in length dramatically within the first hour, and the increase in swelling was again more or less finished within 2 h. In contrast, the control and 20% NCE hydrogels showed almost no change in the swelling ratio following incubation at either 70 °C (Figure 3b) or 5 °C (Figure 3c). We next analyzed the change in gel length in response to temperature by incubating the gels for 2 h at several different temperatures, while increasing the temperature from 5 °C to 70 °C (Figure 3d). The shrinkage of the 20% EMP hydrogel began at around 20 °C and continued until around 50 °C, with the maximum rate of shrinkage occurring in the 40–50 °C range.

### 2.4. Salt Concentration

**Figure 4:**
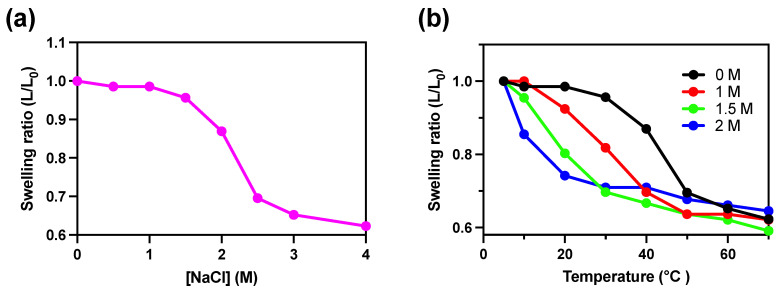
Swelling/shrinking response of 20% EMP hydrogel to temperature in varying salt conditions. (**a**) The swelling ratio of the 20% EMP hydrogel observed after 2 h incubation with buffer containing the indicated concentrations of sodium chloride at 4 °C. (**b**) The temperature dependence of the swelling ratio of the 20% EMP hydrogel in different concentrations of NaCl between 0 and 2 M.

To examine the effects of salt concentration on the shrinking/swelling capacity of the 20% EMP hydrogel, the gel was observed after 2 h of incubation at 4 °C with varying concentrations of sodium chloride (Figure 4a). We observed that increasing the buffer salt concentration led to a decrease in the length of the 20% EMP hydrogel at NaCl concentrations between 1.5 and 2.5 M. These results show that EMP hydrogel shrinks and swells in response to changes in salt concentration as well as changes in temperature. Next, we performed a temperature-dependent shrinking/swelling assay with salt concentrations between 0 M and 2 M (Figure 4b). The results show that the temperature-dependent shrinking-swelling capacity for the of EMP hydrogel changed following the addition of salt, and the LCST of the EMP hydrogel was significantly decreased at increasing salt concentrations. Furthermore, the temperature range in which the EMP hydrogel started to shrink appeared to narrow at increasing salt concentrations.

Our results demonstrate that varying the salt concentration has a substantial impact on the physical properties of hydrogels, which is consistent with previous reports. For example, it has been reported that for a chitosan-poly(vinyl alcohol) hydrogel, varying the buffer and salt concentrations influences the swelling behavior of the gel due to changes in the osmotic pressure both within and outside of the gel [21]. From a theoretical perspective, the influence of buffers and salts on the physical properties of hydrogels, such as swelling, is rooted in the interplay between the electrostatic interactions within the network, as well as ion adsorption [22]. In the case of our EMP hydrogels, the exact mechanisms by which the buffer and salt concentration influence swelling and shrinking are a point of interest that will be explored in future studies. Nevertheless, our results, taken together with previous findings, suggest that the nature of the buffer, its concentration, and the pH of the solution are all important factors that can be exploited to tailor the properties of the hydrogel to a wide range of applications.

### 2.5. Bilayer EMP Hydrogel

**Figure 5:**
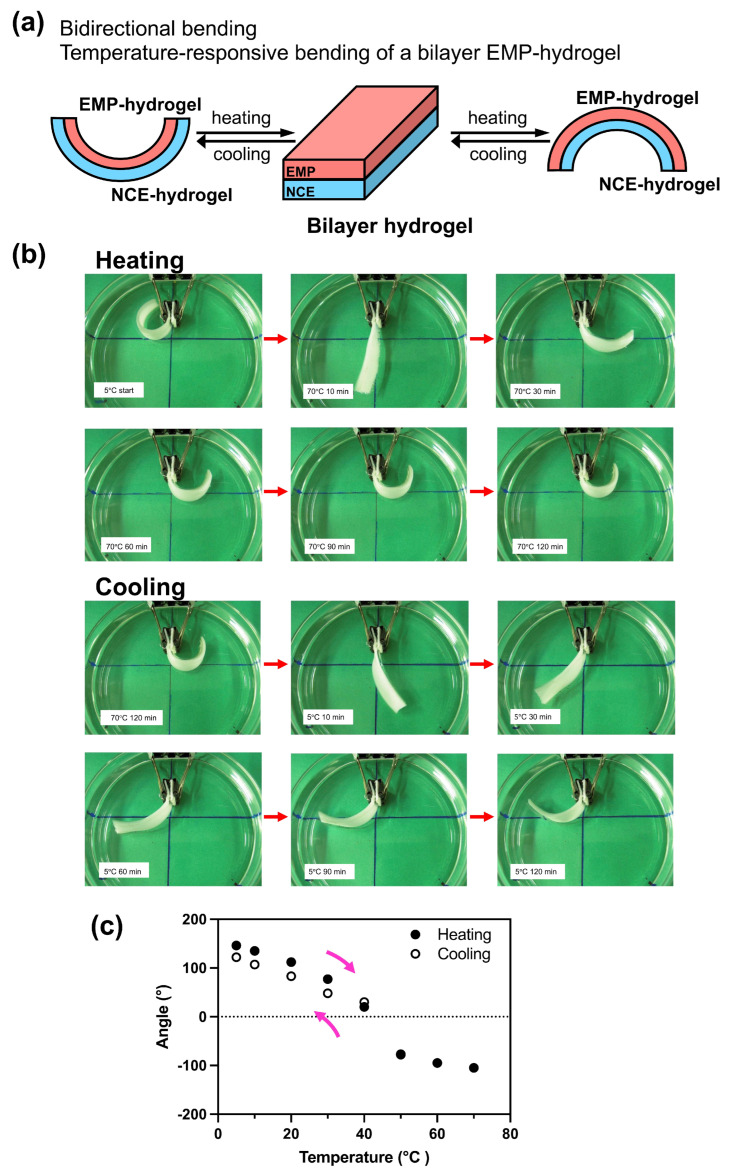
Temperature-responsive bidirectional bending of an EMP-NCE bilayer hydrogel. (**a**) Schematic illustration of the EMP-NCE bilayer hydrogel. (**b**) Photographs of the bilayer hydrogel during heating and cooling periods. For the analysis during heating, the hydrogel was first preincubated at 5 °C for 5 min, and then, the gel was transferred to 70 °C and observed at time points indicated in the upper two panels. For the analysis during cooling, the hydrogel was then incubated first at 70 °C for 2 h; then, the hydrogel was transferred to 5 °C and observed at time points indicated in the lower two panels. (**c**) Bending angle of bilayer hydrogels. The bilayer gel was incubated for 2 h at varying indicated temperatures between 5 °C and 70 °C, and then, the bending angle of the bilayer hydrogel at each temperature after 2 h incubation was measured. The upper arrow indicates heating, and the lower arrow indicates cooling.

Creating hydrogels with varying swelling properties is important for the development of versatile polymers that can be used as smart materials for controlled drug delivery applications due to their ability to respond to changes in stimuli with changes in their volume. With this aim in mind, we next prepared bilayer gels with different shrinking/swelling properties, and analyzed their responses to different temperatures (Figure 5). A 20% EMP hydrogel and a 20% NCE hydrogel prepared at 20 °C were laminated to create an EMP/NCE bilayer gel. To analyze the temperature response of this bilayer gel, the gel was preincubated first at 5 °C for 5 min, and then, at 70 °C for 2 h (Figure 5b). Incubation of the bilayer gel at 5 °C for 12 h caused the two-phase gel to curve toward the EMP hydrogel side. Next, after 12 h of incubation at 5 °C, the EMP/NCE-bilayer gel was then incubated at 70 °C and monitored every 5 min. At 70 °C, the curved gel reverted to its original state and became straight within 10–15 min, and then, curved toward the NCE hydrogel side. After heating at 70 °C, the hybrid gel was incubated again at 5 °C. After this treatment, the hybrid gel that had been curved toward the NCE hydrogel side first straightened, and then, curved in the opposite direction toward the EMP hydrogel side. Interestingly, the hybrid hydrogel returned to the same degree of curvature after 12 h at 5 °C as it displayed at 5 °C before the heating to 70 °C (Figure S1). These results indicate that the EMP bilayer gel underwent reversible expansion and contraction in response to changes in temperature. To further analyze the thermal response of the bilayer gels, the gel was incubated for 2 h at varying temperatures between 5 °C and 70 °C (Figure 5c). For each temperature, the curvature of the gel was determined by calculating the angle of a straight line drawn from the tip of the gel fixation site to the tip of the gel. The results show that the EMP/NCE hybrid gel curved towards the EMP hydrogel side (left side) at temperatures below 40 °C and towards the NCE hydrogel side (right side) at temperatures above 40 °C. Thus, we succeeded in developing functional bilayer gels that can control the curvature direction through temperature changes. Bilayer gels that are responsive to NIPAM and other compositions of hydrogels have been reported [23,24,25]. In one case, it was reported that multi-responsive bilayer hydrogels can bend in both directions in response to temperature and solvent change using NIPAM and poly(N-hydroxyethyl acrylamide) (PHEAm) [26]. However, there are only a few examples of bilayer hydrogels bending in both directions in response to temperature changes. Thus, these newly created smart materials can be potentially used in the fabrication of artificial muscles or other smart structures wherein the bidirectional contraction of the hybrid EMP hydrogels is important.

Our study demonstrates that EMP hydrogels are able to increase their swelling/shrinking capacity by increasing the amount of cross-linking with acrylamide. In this case, the EMP unit of the hydrogel can be selected with a specific and desired number of the elastin repeat sequence. Elastin is known to regulate coacervation ability through its repeat number, and the swelling/shrinking ability of the EMP hydrogel can be controlled by changing the repeat number of the elastin unit of the acryl-EMP or by introducing the functional group into the elastin unit [27,28].

EMPs are characterized by extremely low antigenicity and toxicity to living organisms [15,18]. Although acrylamide polymers were used as the main chains forming the gel network in this study, acrylamide polymers do not play an important role in the development of responsiveness, and monomers can be selected depending on the application. For example, for use in either drug delivery systems or as biological gel actuators, one would develop stimulus-responsive gels suitable for biological applications by selecting polysaccharides and other polymers that exhibit low toxicity and antigenicity.

## 3. Materials and Methods

### 3.1. Plasmid Construction, and Peptide/Protein Purification and Synthesis

The oligonucleotides for the cDNA sequences of (Val-Pro-Gly-Val-Gly)3, 5′-TTGGTGTACCGGGTGTTGGTGTACCGGGTGTTGGTGTACCGGGTGTTGGTGTCCCAGGTG-3′ and 3′-CACAACCACATGGCCCACAACCACATGGCCCACAACCACATGGCCCACAACCACAGGGTC-5′, were annealed and self-ligated to obtain tandem-ligated DNA fragments. The ligated product was then amplified using the following primers: EMP-fwd: 5′-GCTCTAGAGTTCAGGTGTTGGTG-3′, and EMP-rev: 5′-GCTCTAGAGTTCCAGGTGTTGGTG-3′. The DNA fragments were purified, and the resulting cDNA fragments were digested with NotI (New England Biolabs, Beverly, MA, USA) and XhoI (New England Biolabs, Beverly, MA, USA). The Not/XhoI recognition sequence was inserted into the NdeI/BamHI site of the pET-3a vector (Novagen, Madison, WI, USA) using the annealed oligonucleotides (EMP-NdeI and EMP-BamHI). The digested cDNA was inserted into the NotI/XhoI site of the pET-3a vector, in which the NotI/XhoI recognition site was inserted into its NdeI/BamHI site. The resulting plasmid was cloned into the *E. coli* strain BL21(DE3)pLysS (TOYOBO, Osaka, Japan), and the peptide was induced by adding 1 mM isopropyl β-D-thiogalactopyranoside (IPTG, Nacalai Tesque, Kyoto, Japan) for 4 h, followed by protein purification. The obtained cDNA of the EMP unit was also cloned into pCold-IV and purified via His-tag affinity purification (Figure S2). The purified (His)_6_-EMP was measured via ESI-MS, and we found that the number of repeats of (VPGVG) was 37 (ESI analysis calc. for [M+H]^+^: 18,100.2, observed: 18,100.0).

### 3.2. Protein Expression and Purification

The cell pellets from the induction of the pET-3a-EMP plasmid were lysed in lysis buffer (PBS, pH 7.5, 0.02% 2-mercaptoethanol, 0.2% Triton X-100, 500 mM NaCl, 2 mM p-APMSF) using a French press. The lysed cells were centrifuged, and the resulting supernatant was incubated at 50 °C for 15 min, and then, cooled to 4 °C for 15 min to precipitate the EMP protein. After centrifugation at 4 °C and 5000 rpm for 10 min, the supernatant was incubated at 70 °C for 15 min, and then, cooled at 4 °C for 15 min. After centrifugation at 4 °C and 5000 rpm for 10 min, the supernatant was incubated at 50 °C for 15 min. After centrifugation at 50 °C and 6200 rpm for 15 min, the pellet was obtained and resuspended in ice-cold PBS buffer. The samples were then incubated at 4 °C for 15 min and centrifuged at 15,000 rpm for 10 min. The supernatant was collected and the separation via heating/cooling was repeated 2 times (Figure S2).

### 3.3. Synthesis of Acryl-EMP

The elastin peptide was expressed and purified using an *E. coli* expression system, as outlined in Section 3.2. To prepare the thermo-responsive hydrogels, the elastin peptide was conjugated with acrylic acid to prepare acrylated EMPs (acryl-EMP; Scheme S1) as follows. Acrylic acid (5.2 mmol, 2 e.q., Nacalai Tesque, Kyoto, Japan) and 1-ethyl-3-(3-dimethylaminopropyl)carbodiimide hydrochloride (EDC, 2.6 mmol, 1 e.q., Kanto Chemicals, Tokyo, Japan) were dissolved in acetonitrile (Kanto Chemicals, Tokyo, Japan) at room temperature and incubated for 6 h to obtain acrylic anhydride. The acrylic anhydride solution (1.4 mmol, 50 e.q.) in acetonitrile was then added dropwise to a solution of EMP (13 µmol, 1 e.q.) in water, and the resulting mixture was stirred at room temperature for 2 h. The reaction was monitored using a Kaiser test. At the end of the reaction, the mixture was dialyzed into water to remove the acetonitrile and the solution was lyophilized to yield the acryl-EMP.

### 3.4. Temperature-Dependent Turbidity Measurements

A total of 20 µM of Acryl-EMP and 20 µM of EMP protein were dissolved in PBS buffer (137 mM NaCl, 8.1 mM Na_2_HPO_4_, 2.68 mM KCl, 1.47 mM KH_2_PO_4_) or Tris buffer (375 mM Tris-HCl pH 8.8) and OD_400_ (thermal dependence, 1 °C/1 min from 20 °C to 50, 55, 60, or 70 °C), and they were analyzed using JASCO ETC-717 and JASCO V-630 (JASCO, Tokyo, Japan) spectrophotometers.

### 3.5. Preparation of EMP Hydrogel

For the preparation of the 5% and 20% EMP hydrogels, the monomer solvent—acryl-EMP (2.83 µmol, 5% or 11.32 µmol, 20%, respectively) with acrylamide (1.07 µmol, 7.6%, Nacalai Tesque, Kyoto, Japan) and N,N′-methylene bisacrylamide (25.9 µmol, 0.4%, Nacalai Tesque, Kyoto, Japan)—in 1 mL of 375 mM Tris-HCl pH 8.8 buffer were polymerized by adding ammonium persulfate (0.1%, Nacalai Tesque, Kyoto, Japan) and TEMED (0.02%, Kanto Chemicals, Tokyo, Japan). After adding ammonium persulfate and TEMED, the monomer solvent was poured in thin cassettes (1 mm thickness and 90 mm width) and polymerized at 20 °C. Non-crosslinked EMP hydrogels (NCE hydrogels) (5% and 20%) were prepared by using EMP instead of acryl-EMP. For EMP-NCE bilayer hydrogel preparation, the same volume of acryl-EMP monomer solvent was polymerized at 20 °C on top of the NCE hydrogel in the same thin cassettes.

### 3.6. Temperature- and Salt-Dependent Behavior/Response of EMP Hydrogel

Thermal and salt concentration responses were analyzed by incubating the gels in the buffer (375 mM Tris-HCl pH 8.8) with or without varying concentrations of NaCl at the indicated temperature. After incubation, the gel length was measured. For the bilayer gel, the thermal responses were monitored for changes in curvature, which was defined as the angle of a straight line drawn from the center of the tip of the gel fixation site to the tip of the gel.

## 4. Conclusions

In summary, we present the synthesis and functional characterization of hydrogels made from the pentapeptide repeat of elastin. The elastin-mimetic peptides were acrylated and crosslinked to form EMP hydrogels that exhibited a higher capacity for swelling and shrinking, as well as greater sensitivity to temperature, in comparison to non-crosslinked EMP hydrogels. The swelling properties of the EMP hydrogels were also found to be influenced by the salt concentration of the buffer, thereby providing an additional level of control that will be extremely valuable for different biomaterial applications. Finally, a smart bilayer gel that contracts bidirectionally in response to variations in temperature was created based on a hybrid of the EMP hydrogels and NCE hydrogels. These results underscore the importance of chemical modifications to peptides since such an approach could be used to fine-tune the functional properties of these EMP hydrogels. By and large, the results are encouraging and are the first step in maximizing the potential of EMPs. Future work in this area will focus on elucidating the mechanism of response of the gels to different external stimuli. Using the smart EMP hydrogels as scaffolds for artificial muscles or robotics will also be explored.

To date, most stimulus-responsive hydrogels are composed of chemically synthesized polymers, such as NIPAM. To apply stimulus-responsive hydrogels in biomaterials, there is an important need to develop materials using non-toxic biologically derived components. In this study, we demonstrated that biologically derived hydrogels composed of an elastin-mimetic polypeptide displayed bidirectional bending in response to temperature changes. Determining the optimal conditions for preparing EMP hydrogels requires thorough examinations of several factors, including the percentage of acryl-EMP, the affected different buffer salts, and the precise changes in temperature.

## Data Availability

All data supporting this study are contained within the article.

## Supplementary Materials

The following supporting information can be downloaded at: https://www.mdpi.com/article/10.3390/molecules28135274/s1, Scheme S1: Synthesis of acryl-EMP, Figure S1: Photographs of the EMP-NCE bilayer hydrogel during cooling periods, Figure S2: SDS-PAGE analysis of purified EMP and (His)_6_-EMP.

## Sample Availability

Samples of the compounds are not available from the authors.

## Abbreviations

The abbreviations used are as follows: EMP, elastin-mimetic polypeptide; NCE, non-crosslinked EMP; EDC, 1-ethyl-3-(3-dimethylaminopropyl)carbodiimide hydrochloride; LCST, lower critical solution temperature; PHEAm, poly(N-hydroxyethyl acrylamide); PNIPAM, poly(N-isopropylacrylamide); PVME, polyvinyl methyl ether; IPTG, isopropyl β-D-thiogalactopyranoside.
