# Supplementary material for: Bilayer Hydrogel Composed of Elastin-Mimetic Polypeptides as a Bio-Actuator with Bidirectional and Reversible Bending Behaviors
Source: Molecules. 2023 Jul 7;28(13):5274. doi: 10.3390/molecules28135274 (PMC10343461; doi:10.3390/molecules28135274)
Supplement: Supplementary file 1 [file molecules-28-05274-s001.zip › molecules-2480541-supplementary.pdf]

Supplementary Materials

Bilayer Hydrogel Composed of Elastin-Mimetic Polypeptides as a Bio-Actuator with Bidirectional and Reversible Bending Behaviors

Rui Kamada [1], Hiromitsu Miyazaki [1], Jose Isagani B. Janairo [2], Yoshiro Chuman [3] and Kazuyasu Sakaguchi [1,*]

[1] Laboratory of Biological Chemistry, Department of Chemistry, Faculty of Science, Hokkaido University, Sapporo 060-0810, Japan; kamadar@sci.hokudai.ac.jp (R.K.)
[2] Department of Biology, College of Science, De La Salle University, Manila 0922, Philippines; jose.isagani.janairo@dlsu.edu.ph
[3] Laboratory of Biological Chemistry, Department of Chemistry, Faculty of Science, Niigata University, Niigata 950-2181, Japan; chuman@chem.sc.niigata-u.ac.jp
* Correspondence: kazuyasu@sci.hokudai.ac.jp

Contents:

Scheme S1. Synthesis of acryl-EMP.
Figure S1. Photographs of the EMP-NCE bilayer-hydrogel during cooling periods. After heating at 70°C, the hydrogel was incubated at 5°C for the indicated time periods.
Figure S2. SDS-PAGE analysis of purified EMP and $(His)_6$-EMP.

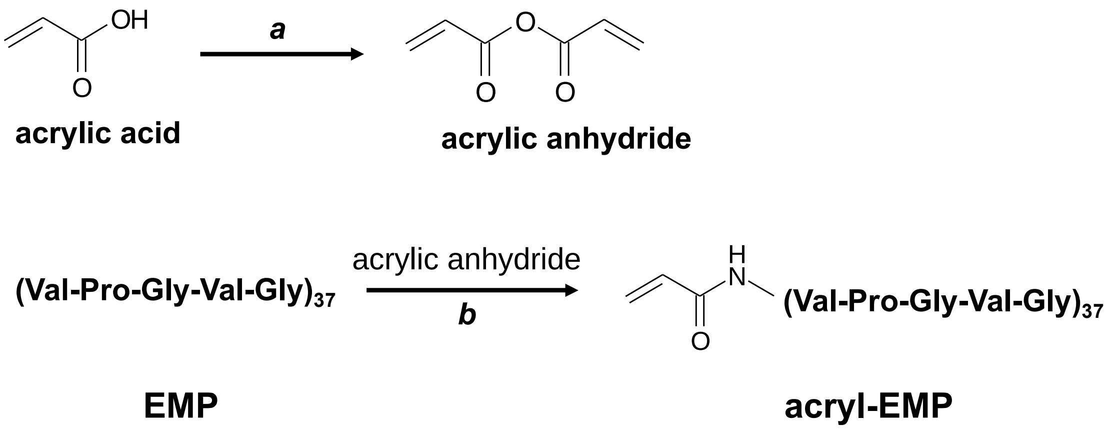

Scheme S1. Synthesis of acryl-EMP. Reagents and conditions: (a) 1-ethyl-3-(3-dimethylaminopropyl)carbodiimide hydrochloride (EDC), acetonitrile, rt for 6 h. (b) acetonitrile, rt for 2 h.

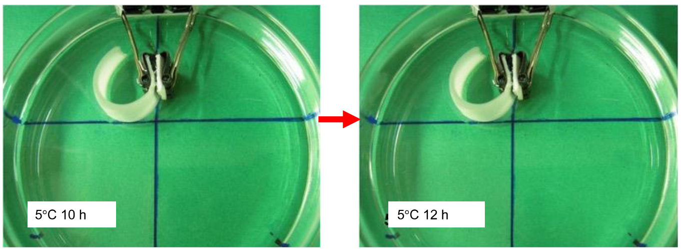

Figure S1. Photographs of the EMP-NCE bilayer-hydrogel during coolingperiods. After heating at 70°C, the hydrogel was incubated at 5°C for the indicated time periods.

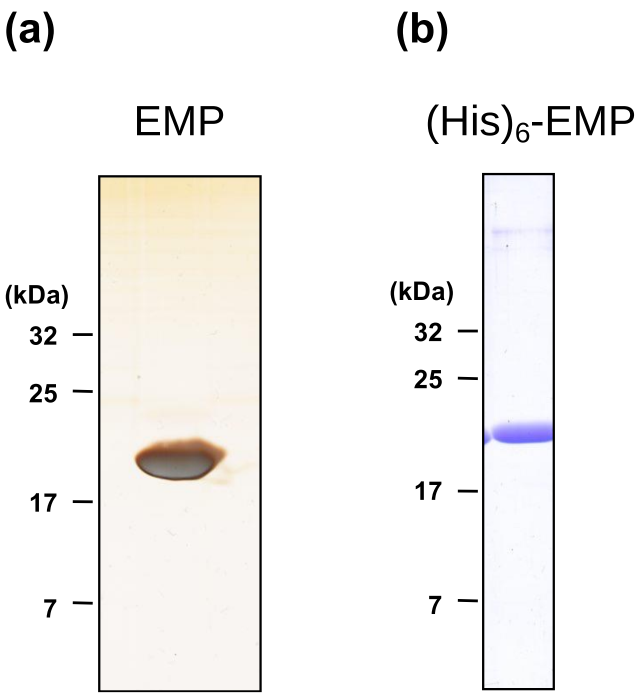

Figure S2. SDS-PAGE analysis of the purified EMP and $(His)_6$-EMP. The gels were visualized by silver staining for EMP (a) or CBB-R250 staining for $(His)_6$-EMP (b). The silver staining of SDS-PAGE showed that the EMP was highly purified.
